# Kataegis associated mutational processes linked to adverse prostate cancer presentation in African men

**DOI:** 10.21203/rs.3.rs-4597464/v1

**Published:** 2024-06-28

**Authors:** Vanessa Hayes, Jue Jiang, Avraam Tapinos, Ruotian Huang, Riana Bornman, Phillip Stricker, Shingai Mutambirwa, David Wedge, Weerachai Jaratlerdsiri

**Affiliations:** University of Sydney; Garvan Institute of Medical Research; University of Manchester; University of Sydney; University of Pretoria; St. Vincent’s Hospital; Sefako Makgatho Health Science University; University of Manchester; University of Sydney

## Abstract

Kataegis, the focal hypermutation of single base substitutions (SBS) in tumour genomes, has received little attention with respect to prostate cancer (PCa) associated molecular and clinical features. Most notably, data is lacking with regards to this tumour evolutionary phenomenon and PCa racial disparities, with African men disproportionately impacted. Here through comparison between African (n = 109) and non-African (n = 79) whole genome sequenced treatment naïve primary tumours, using a single analytical workflow we assessed for shared and unique features of kataegis. Linking kataegis to aggressive presentation, structural variant burden and copy number loss, we attributed APOBEC3 activity through higher rates of SBS2 to high-risk African tumours. While kataegis positive African patients presented with elevated prostate specific antigen levels, their tumours showed evolutionary unique trajectories marked by increased subclonal and structural variant-independent kataegis. The potential to exacerbate tumour heterogeneity emphases the significance of continued exploration of biological behaviours and environmental exposures for African patients.

## Introduction

Prostate cancer (PCa) is the most frequently diagnosed male cancer in most regions of the world, yet men of African ancestry and particularly from Sub-Saharan Africa, are disproportionately impacted^1^. Mortality rates of PCa are highest across Sub-Saharan Africa and the Caribbean, with the highest rates reported for southern Africa (29.7 age-standardised per 100,000 males) although having a lower incident rate (59.9) than Australia and New Zealand (78.1). Conversely, both incidence and mortality rates are lowest across the Asian diaspora of nations, including Eastern Asia with rates of 15.3 and 3.8, respectively. Although this disparity may be attributed to diminished access to PCa screening and medical resources, or exposure to yet unknown geographic risk factors, studies out of the United States have shown that African American men are at greatest risk for aggressive disease presentation and associated lethality after accounting for non-genetic factors^2, 3^. Besides genetic susceptibility, these studies have alluded to both biological and genomic contributions.

While more research has been urged to address health security of African men^4^ at greatest risk of PCa adversity, representative prostate tumour and patient paired blood whole genome sequencing (WGS) data is lacking. This is evidenced by the most recent release (Data Release 40.0 on March 29, 2024) for the US-based The Cancer Genome Atlas (TCGA) where of 500 PCa cases, 415 are European ancestral (83%) and only 58 African (11.6%) and 12 of Asian ancestry (2.4%). Also, the largest Pan-Cancer Analysis of Whole Genomes (PCAWG) study on 38 cancer types from 2,658 patients, lacked data for Sub-Saharan Africa^5^. Establishing the Southern African Prostate Cancer Study (SAPCS)^6^ has provided this team the opportunity to begin to address broader African inclusivity. Reporting not only elevated aggressive disease presentation compared to black Americans^7^, additionally we revealed both germline and somatic genomic disparities through direct European ancestral comparative analyses. Specifically, we have shown southern African specific rare^8^ and common^9^ genetic risk variants, higher levels of somatic short variants, unique genomic complexity and mutational signatures, distinctive somatic driver events along evolutionary periods, and a unique molecular taxonomy^10, 11^. Additionally, both germline and somatic variance has been determined at the single-gene^12^, mitogenome^13^ and chromosomal level^14^. Observing no ancestral differences in the total number of structural variants (SVs) acquired during prostate tumourigenesis, yet a significant difference in the total number of small variants, including single nucleotide variants (SNVs) and insertions deletions (indels, < 50 bases)^10^, here we turn our attention to kataegis, an intermediary linking these small and larger variants.

Kataegis was first identified in breast cancer^15^ and later observed in many cancer types, mostly commonly bladder, lung, and skin-melanoma^5, 16, 17^. A Greek word meaning thunderstorm, kataegis describes the focal hypermutation phenomenon in cancer genomes, which manifests as a cluster of closely distributed SNVs with the following characteristics: kataegic SNVs are usually C > T and C > G transitions in *cis* with T at the 5’ flanking nucleotide and co-localise with breakpoints of SVs^15^. Kataegic SNVs likely originated simultaneously from exposed single strand DNAs (ssDNA) by APOBEC3A and APOBEC3B cytidine deaminases, as evidenced by yeast experiments^18^ and through association with APOBEC genes expression^5^. Being less common in PCa compared to other cancer types^5^, kataegis has received little to no focus^19, 20, 21^. In turn, kataegis remains unexplored with respect to ancestrally derived tumour genome and clinical disparities, Specifically aggressive disease in African men. Building on our previous comprehensive PCa study^10^, we explored the contribution of kataegis through interrogation of 188 WGS prostate tumours (166 published) from 109 African (black South African) men, allowing for direct comparative analysis with technically and analytically matched tumour genome profiles from 57 European and an additional 22 (this study) Asian men. Besides genomic and clinical associations, we provide further evidence for ancestral differences in evolutionary timing and mutational process.

## Results

### Prevalence and distribution of kataegis by ancestry

Using our multi-ethnic cohort, patient ancestry was confirmed using genetic substructure analyses (see Methods). Specifically, 109 patients presented with over 86% African ancestry, 56 patients with over 90% European ancestry and a single patient with 74%, while 22 patients presented with over 89.6% Asian ancestry (Table 1). While no alternative whole-genome African-relevant validation data was available, we expanded our interrogation of non-African validation data to include 296 European^22, 23^ and 207 Asian^24^ patients obtained from International Cancer Genome Consortium (ICGC) data portal and previous publications. With PCa aggressiveness defined by International Society of Urological Pathology (ISUP) Grade Group (GG), it should be appreciated that to better match our African cohort (72/109, 66% GG4/5), patient selection for our European study was biased towards untreated advanced disease (49/57, 86% GG4/5) and as such is notably different to public European data (3/296, 1% GG4/5). While small, our Asian study (7/22, 32% GG4/5) is more comparable to published Asian data (94/207, 45% GG4/5). However, to achieve statistical power we used a cut-off of ISUP GG ≥ 3 to define high-risk (HR) over low-risk (LR) tumours derived from histopathological Gleason score at diagnosis (black South Africans) or surgery (European and Asian Australians).

**Figure 1:**
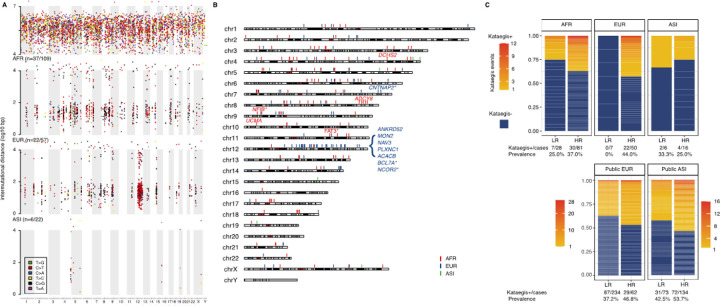
Distribution and prevalence of kataegis in PCa derived from a multi-ethnic cohort of 188 patients. **A.** Distribution of clustered kataegic SNVs of PCa patients by ethnicity, including African (AFR, 37/109), European (EUR, 22/57) and Asian (ASI, 6/22). The background random SNVs (inter-mutational distance > 10kb) were extracted from the single European patient with intensive burden of kataegis events biased to chromosome 12. **B**. Kataegis distribution labelled on cytobands for patients of African (red), European (blue) and Asian (green) ancestries. Labelled genes include genes with missenses kataegic SNVs, and genes found in Cancer Gene Census (CGC) gene list with an asterisk further noted. **C.** Prevalence of kataegis by patient ancestral identifier and risk level defined as low-risk (LR) for GG1 and GG2, and high-risk (HR) for GG3 to GG5 clinicopathological presentation. While kataegis negative (kataegis−) tumours are in dark blue, the yellow to red gradient for positive (kataegis+) tumours represents the number of kataegis events from smallest to largest. The public validation cohorts included data from 296 EUR and 207 ASI PCa patients defined by clinical presentation.

As the largest study on kataegis to date^5^ and allowing for direct comparative analyses, we followed the PCAWG consortium kataegis identification method which allows at least four consecutive SNVs with inter-mutational distance less than 1 kbp depending on mutational burden (see Methods). From the studied cohort (n = 188), we identified 249 kataegis events (Extended Data Table S1) in 65 cases (34.6%), representing 37/109 (33.9%) African, 22/57 (38.6%) European and 6/22 (27.3%) Asian men (Fig. 1A), with tumour genome wide distribution (Fig. 1B). From published data, we identified 321 and 297 kataegis events in 116/296 (39.2%) European and 103/207 (49.8%) Asian patients, respectively (Fig. 1C). Determining the size, number, and distribution of kataegis events across our multi-ethnic cohort, we observed kataegis of small size and span, and low frequency in PCa, as previously described^5^. A median kataegis event consists of six kataegic SNVs (range 4–33) spanning 2.7 kbp (range, 0.2–35.3). Irrespective of ancestry, kataegis positive tumours presented with occasional kataegis events (median 2, range 1–12), except for a single European derived hyper-kataegic outlier (45 events). Consistent with previous reports^25^, the regions of kataegis events were private. Approximately 40% (725/1823) of the kataegic SNVs spanned within genes or in the regulatory regions of genes, the majority within introns (611, Extended Data Table S2) and including a few (11, Extended Data Table S3) missense variants in nine genes from three African and two European derived tumours, namely *ANKRD52, MON2, NAV3, PLXNC1, ACACB, ADCY8, UCMA, DCHS2*, and notably the PCa oncogene *NCOR2* (Fig. 1B). Additionally, intronic kataegis events were observed spanning known driver genes of other cancer types, gathered in the most recent Cancer Gene Census (CGC, v98), Specifically *PCAT1, BCL7A, CNTNAP2, FAT3*, and *NFIB* from two African and two European derived tumours.

### Kataegis related to cancer aggressiveness, genomic instability, and clinical presentation

**Figure 2:**
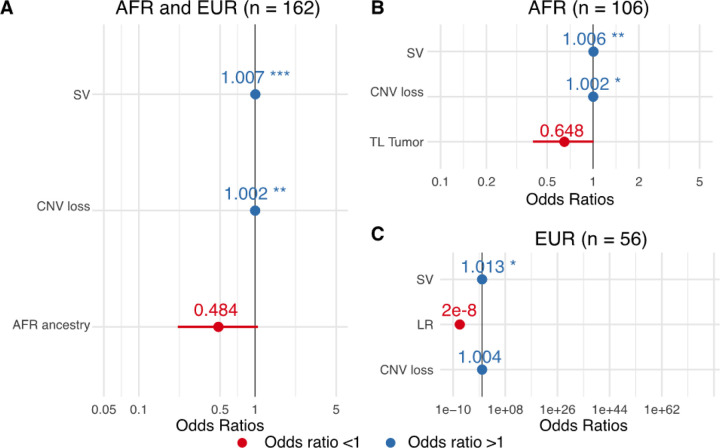
Odds ratios of logistic models selected contributing factors for kataegis presence. **A**. The contributing factors of model fitting African (AFR) and European (EUR) derived tumours (n = 162). The ancestry factor used European as a reference. **B**. The contributing factors of model fitting African derived tumours (n = 106) and **C** European derived tumours (n = 56). The clinicopathological presentation factor use low-risk (LR, GG1/2) as a reference. Negative correlation is indicated by odds ratio <1, shown in red colour, Positive correlation is indicated by odds ratio > 1, shown in blue colour. Contributing factors selected by model are not all significant. If significant, we labelled significant level descriptors: *, *P*-value < 0.05; **,*P*-value < 0.01; ***, *P*-value < 0.001.

As kataegis positive genomes presented with elevated small and structural variants (FDRs = 7e-5–0.045, Wilcoxon rank sum test; Extended Data Fig. 1), we used generalised linear models (GLM) to investigate for kataegis associated tumour molecular features. Although tumour mutational burden (TMB) was associated with kataegis (Extended Data Fig. 1), it was excluded in the model selection as TMB and SV burden were dependent (correlation = 0.66). Through GLM built from African and European data, we observed tumours susceptible to the presence of kataegis exhibited higher SV burden and more regions of copy number loss (logistic regression model, *P*-values = 0.0005–0.002; Fig. 2A). Ancestry also showed contribution to the presence kataegis as selected in the model, although not significant (*P*-value = 0.07, Fig. 2A), which suggests tumours of African ancestry were less likely to present kataegis compared to European ancestry. Due to the potential impact of ancestry, we further analysed models with African-only and European-only data. While a link to tumour telomere length was suggested for African data and a link to risk-level for European data, significance was not achieved (*P*-values = 0.061 and 0.994, respectively; Fig. 2B, C). These support the necessity of continued analyses on multi-ethnic studies. Apart from genomic factors, prostate-specific antigen (PSA) levels at diagnosis were higher in kataegis positive versus negative tumours for African patients with HR PCa only (median,100 *vs* 49.7 ng/mL, Wilcoxon’s rank-sum test, False discovery rate, FDR = 0.04; Extended Data Fig. 2). The latter suggests a possible link between kataegis and clinical presentation for African patients.

Further focusing on kataegis burden of PCa, we found positive association for somatic SV burden (negative binomial model, *P*-value = 8e-15), as described by PCAWG^5^. An additional positive association with HR presentation (*P*-value = 0.01) was observed, which was observed in a subtype of breast cancer^26^. More kataegis events in HR PCa was also shown in the public sourced Asian cohort (2 *vs* 1 median events, Wilcoxon’s rank-sum test, *P*-value = 0.02). This finding implicates burden of kataegis as a biomarker of PCa aggressiveness and genomic instability, irrespective of patient ancestry.

### Varying evolution of kataegis events between ancestries

Tumour molecular features have been shown to be dynamic along tumour evolution^10^. Linking kataegis with molecular features, we further interrogated evolutionary timing of kataegis between the ancestries. Following the timing categories described by PCAWG consortium^27^, we estimated the evolutionary timing of clonal kataegis (including early, late, and unspecified) and subclonal kataegis. However, rather than calling kataegis from multi-epoch SNVs as performed by PCAWG consortium, we identified kataegis from SNVs raised from the same epoch to improve the accuracy (see Methods). Consequently, we observed pairs of kataegis regionally overlapped but raised from different epochs in three HR tumours (2 European, 1 African, Extended Data Table S4).

**Figure 3:**
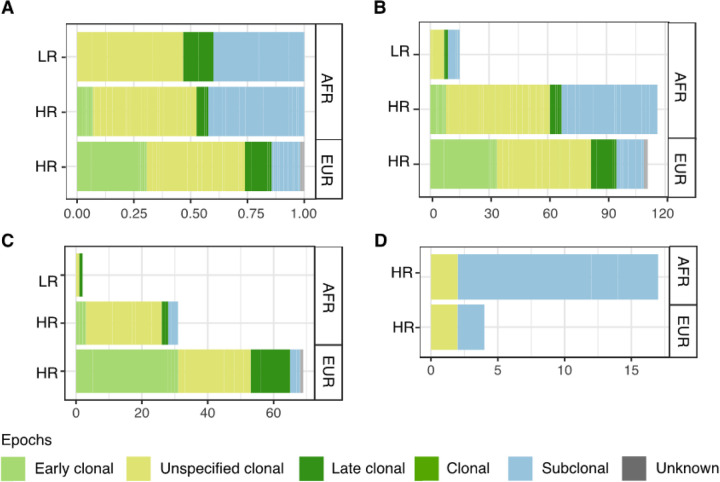
Evolution of kataegis events in risk levels of prostate tumours derived from African (AFR), European (EUR) (n = 166). Evolution of kataegis events by ancestry and risk-level and shown as **A** proportion and **B** number of different epochs. The risk level of tumour was defined as low-risk (LR) for GG1/2, and high-risk (HR) for GG3–5 clinicopathological presentation. **C** and **D** shows evolution of kataegis events shows co-occurring with clonal mutations, and subclonal mutations in *TP53*or *ATRX*, respectively.

Observing both clonal and subclonal events, clonal kataegis is predominant in our study (69.9%, 174/249), with same trend also observed in PCa reported by PCAWG, although not showing significance as is the case for several other cancer types^5^. While proportions of genome-wide SNVs per epoch were similar between ancestries (Extended Data Fig. 3A), evolution of kataegis varied. Compared to European derived tumours, African tumours showed more subclonal kataegis, irrespective of risk-level (odds ratios of clonal/subclonal kataegis, African derived LR = 1.5, African derived HR = 1.4, European derived HR = 6.8, Fisher’s exact test on HR, *P*-value = 7e-7; Fig. 3A, B). Conversely, early clonal kataegis was biased towards European derived HR tumours, although not significant (odds ratios of early clonal/late clonal kataegis, European = 2.6 *vs* African = 1.3, Fisher’s exact test, *P*-value = 0.3). Asian derived tumours showed balanced proportions of clonal and subclonal kataegis events (Extended Data Fig. 3B, C). Associating kataegis with genomic instability, the varying ancestrally relevant timing of kataegis suggests loss of genomic stability as a late rather than early event in African versus European prostate tumorigenesis and may be implicated in giving rise of subclones in African patients.

### Kataegis events linking molecular subtypes and clinical implication by ancestry

As our previously reported global mutational subtypes (GMS), an ancestrally defined PCa molecular taxonomy which showed different evolutionary trajectories and associated driver genes^10^, we examined for possible associations of kataegis with GMS. We found kataegis to be biased towards GMS-C (Prevalence, GMS-C = 59.1% *vs* other GMS = 31.9%, Fisher’s exact test, *P*-value = 0.02; Extended Data Fig. 4), an African and European specific subtype, correlated with early stage driver gene mutation and associated homozygous loss in two cell-cycle checkpoint genes, *TP53* and *ATRX*, respectively^10^. Notably, we found concurrence of kataegis and mutations in *TP53* or *ATRX* for both African and European tumours (Fisher’s exact test, FDRs = 0.003 for African and 0.02 for European; Extended Data Fig. 5), which we further validated in the public European dataset (Fisher’s exact test, *P*-value = 0.003). Furthermore, we found kataegis and checkpoint *TP53* or *ATRX* gene mutations to mostly co-occur during tumour evolution, as observed genomes exhibited clonal mutations in checkpoint genes were enriched with early clonal kataegis (Fisher’s exact test, *P*-value = 1.88e-12; Fig. 3C, D). In contrast, the association was not identified for Asians in the studied or public cohort (*P*-values > 0.44, Extended Data Fig. 5), which is in concordance with our previous observation of a lack of GMS-C representation in Asian derived tumours^10^.

**Figure 4:**
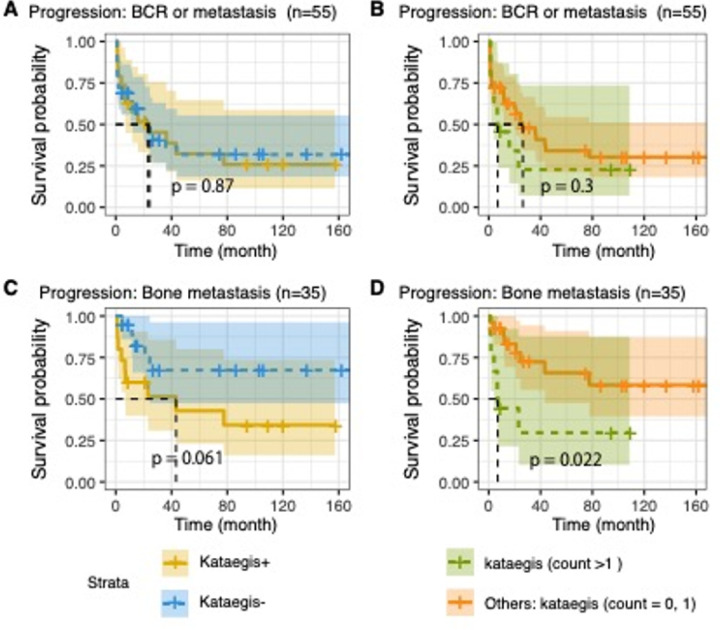
Kaplan-Meier survival estimates correlating presence of kataegis with log-rank test on European and Asian patients with high-risk (HR) tumour by clinical follow-up (time in months). Results of estimated progression, defined as biochemical relapse (BCR) and/or metastasis, with comparison of **A**. patients with kataegis positives (+, n = 19) versuskataegis negatives (−, n = 36), and **B**. patients with multiple kataegis (kataegis events > 1, n = 11) versus other patients having one or no kataegis events (n = 44). HR tumours were defined as having GG3–5 clinicopathological presentation.Results of estimated clinical progression defined as bone metastasis, excluding BCR patients without bone metastasis are in **C**. and **D**. comparing kataegis presence (kataegis+, n = 15 *versus* kataegis−, n = 20), and elevated kataegis (multiple kataegis, n = 9, *versus* one or no kataegis events, n = 26), respectively.

Having linked kataegis with GMS-C prominent features, we further explored for clinical implications. Whilst GMS-C represents worse clinical outcome than the ancestrally ‘universal’ (all ancestries) GMS-A^10^, kataegis showed higher prevalence in GMS-C than GMS-A tumours (59.1% *vs* 32.3%, Fisher’s exact test, *P*-value = 0.02). We further performed Kaplan-Meier estimates for Eurasian patients with HR tumours. For HR tumours in the presence of kataegis or multiple kataegis (events > 1), we observed no difference in cancer progression, defined as PSA measurable biochemical relapse (BCR) and/or metastasis (Log-rank test, *P*-values > 0.3; Fig. 4A, B). Further we confined the cancer progression with bone metastasis. Although not significant for presence of kataegis, susceptibility to bone metastasis was observed with significance for elevated kataegis burden (Log-rank test, *P*-values = 0.061, 0.022, respectively; Fig. 4C, D). Our findings were further validated in European public data associating multiple kataegis positives with metastasis for LR tumours (Log-rank test, *P*-value = 0.01, Extended Data Fig. 6). However, the potential clinical impact of elevated kataegis for African patients required more clinical data within the southern African cohort.

### Kataegis associated mutational signatures and PCa aggressiveness by ancestry

**Figure 5:**
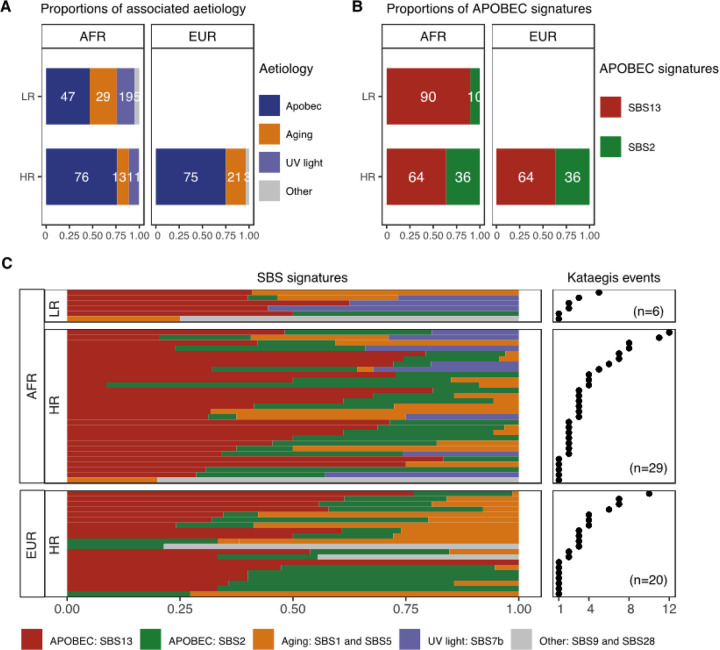
Single-base substitution (SBS) and associated aetiology contributing to kataegis identified in PCa. **A** Proportion of related aetiology contributing to kataegis grouped by risk level and ancestry. APOBEC aetiology shows proportion of SBS2 and SBS13. Aging aetiology shows proportions of SBS1 and SBS5. Ultraviolet (UV) light exposure shows proportion of SBS7b. Others show proportions of SBS9 and SBS28. Genomes are grouped by ancestry, African (AFR) or European (EUR), and by risk-level, defined as low-risk (LR) for GG1/2, and high-risk (HR) for GG3–5 clinicopathological presentation. A hyper-kataegic tumour was excluded. **B**. Proportion of APOBEC signatures SBS2 and SBS13. **C** proportions of APOBEC-related signatures SBS2 and SBS13 and other associated aetiology contributed to kataegis per tumour. Each row represents a tumour, ordered by kataegis burden shown as dot on the right, with number of cases labelled in brackets. APOBEC related signatures SBS2 and SBS13 are in different colour, while other signatures are in the same colour as in **A**.

Previous studies^17, 18, 28^, including PCAWG^5^, have associated kataegis with APOBEC3 and single-base substitution (SBS) mutational signatures SBS2 and SBS13. Correlating aetiologies of kataegis with SBS mutational signatures, we found a median of 82% kataegis events to be attributed to APOBEC signatures in HR tumours, irrespective of patient ancestry (African, European = 82%, public European, Asian = 86%; Fig. 5A, Extended Data Fig. 7). Our findings concurring with PCAWG data (81.7%)^5^. Observing a trend towards more attribution to APOBEC signatures for African derived HR over LR tumours (APOBEC signatures percentage, HR = 82% *vs* LR = 46%, Wilcoxon’s rank-sum test, *P*-value = 0.07; Fig. 5A), while SBS13 over SBS2 was significantly represented in HR African tumours (median against APOBEC signatures, SBS13% = 68% *vs* SBS2 = 32%, Wilcoxon’s rank-sum test, *P*-value = 1e-5; Fig. 5B), relative to SBS13, SBS2 was significantly associated HR over LR African tumours (medians of SBS2 against APOBEC signatures, HR = 32% *vs* LR = 0%, Wilcoxon’s rank-sum test, *P*-value = 0.04). However, the proportion of APOBEC signatures didn’t correlate with kataegis burden (correlation = 0.08; Fig. 5C). Small mutations in genes *APOBEC3A* and *APOBEC3B* were infrequent in the studied cohort and showed no association with kataegis presence (Extended Data Fig. 5). Conclusive statements for Asians were challenging, because of only six kataegis positives (Extended Data Fig. 8). Apart from APOBEC signatures, 18% of kataegis events could be attributed to aging related signatures SBS1 and SBS5, ultraviolet light exposure-related SBS7b, polymerase-related SBS9, and unknown aetiology SBS29.

### Kataegis and genomic rearrangements in high-risk PCa

**Figure 6:**
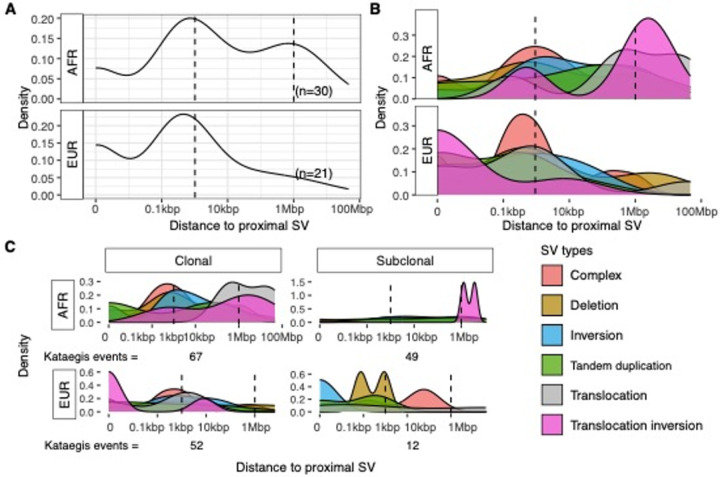
Distance between kataegis and their proximal SVs in African (AFR, n=30) and European (EUR, n=21) derived high-risk (HR) tumours. HR tumour was defined as for having GG3–5 clinicopathological presentation. **A**. Distribution of distances between kataegis and their proximal SVs. Numbers of subjects of each group are labelled in brackets. A hyper-kataegic tumour was excluded. **B**. Distance between kataegis and their proximal SVs of each SV type. **C**. Distance between clonal and subclonal kataegis and their proximal SVs of each SV type. Numbers of clonal and subclonal kataegis of each ancestry are labelled on the bottom.

Having associated the presence of kataegis with SV burden, together with PCAWG showed kataegis colocalised with SVs across tumour types^5^, we investigated for the distribution of SVs proximal to kataegis in African and European patients with HR clinical presentation. Tumours of LR and/or derived from Asian patients were excluded due to small sample sizes, as well as the single hyper-kataegic European-derived tumour. Observing peaks of proximal SVs around 1kbp distance to kataegis events for both ancestries (Fig. 6A), we tested the enrichment of kataegis within 10 kbp of proximal SVs and found significance compared to simulations (see methods; Fisher’s exact test, FDR, African = 2e-22, European = 3e-22). In particular, 48.9% (89/182) kataegis situated within 1 kbp of SV breakends, including 26 spanning SV breakends (Fig. 6A). Distinguishing SVs by type, we found deletions and complex rearrangements to be prominently associated with kataegis for both African and European tumours (Fig. 6B), again concurring with PCAWG data^5^. Most complex rearrangements were chromothripsis (113/117) which also showed concurrence with kataegis events (Fisher’s exact test, *P*-value = 2e-13; Extended Fig. 5).

**Figure 7:**
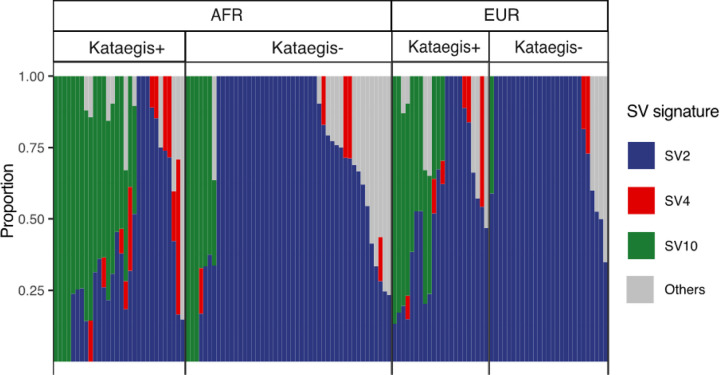
Proportions of SV signatures that differentiated between kataegis positive (+) and negative (−) contributed to the whole genome SVs of high-risk PCa derived from African (AFR, n = 77) and European (EUR, n = 49). SV2, SV4, and SV10 are shown in colours while other SVs are in grey. Each column represents a tumour, ordered by SV10 and SV2. High-risk (HR) tumour was defined as for having GG3–5 clinicopathological presentation.

Another farther and relatively smaller peak around 1 Mbp distance was shown in African derived tumours (Fig. 6A), which was also testified by compared to simulated regions (Fisher’s exact test, FDR, African = 3e-5, European = 0.2, distance region between 0.1 Mbp and 10 Mbp). Proximal SVs around 1 Mbp distance in African derived tumours were mainly translocation and translocation inversions which were, in contrast, enriched at 1 kbp in European tumours (Fig. 6B, Extended Data Fig. 9). Furthermore, in African derived tumours, we observed distant translocations occurring mostly with clonal kataegis, and translocation inversions with both clonal and subclonal kataegis (Fig. 6C). As the distribution of proximal SV types varied along evolutionary timing, we used COSMIC SV signatures^29^ to analyse mutational processes (Extended Data Fig. 10). In both ancestries, kataegis positive tumours showed more SV4 and SV10 presence and lower rates for the predominant SV2 signature (Wilcoxon’s rank sum test, FDRs = 8.5e-4; Fisher’s exact test, FDRs = 6.5e-5–9.9e-3; Fig. 7). According to the COSMIC database, simple translocations and clustered translocations are the primary components of SV2 and SV4, respectively, while other simple rearrangements compose SV10. Consequently, kataegis positive genomes harboured more clustered translocations and simple SVs of other types, with fewer simple translocations accordingly.

## Discussion

Kataegis, or focal hypermutation, has largely been overlooked in PCa, especially with regards to African ancestry and associated aggressive disease presentation. Merging published^10^ and new multi-ethnic WGS data, including data from Sub-Saharan Africa, we observed universal features that are shared among ancestries, and are also in keeping with observations of kataegis of PCa reported by PCAWG^5^. Observed for other cancer types, kataegis associations with cancer aggressiveness^26, 30^ and evolutionary timing^16^, were verified in PCa. Leveraging our unique dataset with 68.1% (128/188) highly advanced GG4 or GG5 tumours, kataegis associations with SBS2, PSA level were observed for African patients. Elevated burden of kataegis was further associated with bone metastasis in European and Asian patients.

Facilitated by our African-inclusive study design, we used kataegis as a probe to report different mutational processes among ancestries. Kataegis raised in both clone and subclone with similar amount in African derived tumours, regardless of clinicopathological presentation, while kataegis originated mostly in the clonal epoch, especially the early clonal epoch for European patients with aggressive disease. Given that kataegis is associated with tumour chromosomal instability observed in this study and indicated by PCAWG^30^, we speculate that kataegis may be implicated throughout tumourigenesis in Africans, contributing to tumour diversification and associated genomic heterogeneity. In contrast, for European derived tumours, kataegis occurs at early epochs and may be implicated in cancer initiation. Another ancestral disparity included the bimodal distribution between kataegis and their proximal SV in African prostate tumours, which observed for breast cancer^31^, was less noticeable for European patients. The distribution indicates the co-existence of comparable amount of independent kataegis, and SV-associated kataegis for Africans, while the former was of relatively low proportion for Europeans.

**Figure 8:**
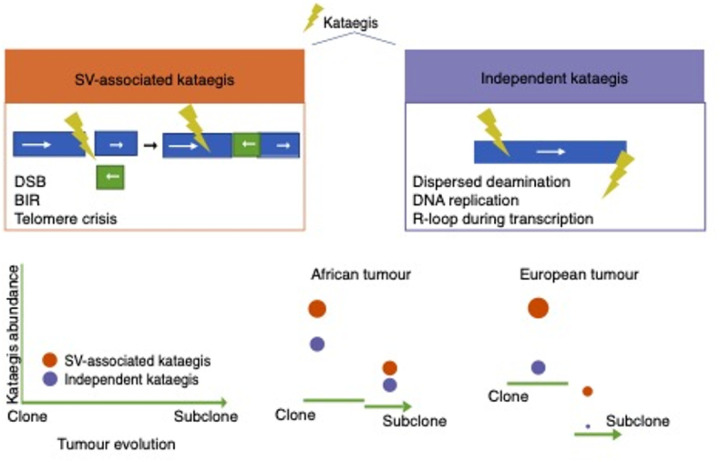
Proposed ancestrally distinct kataegis evolutionary trajectories in high-risk prostate tumourogenesis. The yellow thunder represents kataegis. Proposing two types of kataegis; **SV-associated kataegis** (brown) arises during DNA repair after double-strand breaks (DSBs), break induced replication (BIR), and telomere crisis, while **independent kataegis** (blue) is raised from dispersed APOBEC3 activity, lagging strand of DNA replication, and R-loop during transcription. Furthermore, we propose that these two types of kateagis originate at different rates (indicated by circle size and height) during the evolution, from clone to subclone, of high-risk (GG3–5) African versus European derived prostate tumours.

Although the independent kataegis and SV-associated kataegis were mainly attributed to APOBEC3 deamination of cytosines, we speculate that the underlying mutational process may differ (Fig. 8). Independent kataegis may consist of dispersed APOBEC3 induced mutations, APOBEC3 deamination on R loops in transcription bubbles and on the lagging strand of the DNA replication fork^31, 32^. The resulting uracils may further trigger DNA-repair process^31^, leading to SV-associated kataegis, which could explain the two sequential kataegis around the same region observed in three aggressive tumours. Notably, the amount of independent kataegis events in African derived tumours was maintained throughout cancer development, which is in keeping with the observation of elevated TMB in African over non-Africans tumours^10^. While we haven’t observed a direct link between APOBEC3A/B gene mutations and kataegis, future transcription studies are required to confirm the elevated off-targeted APOBEC3 activity in African derived tumours.

Proximally located SV-associated kataegis may have originated from exposed ssDNA during DNA repair after double-strand breaks (DSBs) and break induced replication (BIR)^33, 34, 35^. Additionally, chromothripsis-associated kataegis has been proposed to be the consequence of telomere crisis in a cell by-passing the checkpoint due to the dysfunction of cell-cycle checkpoints genes^36^, such as *TP53* and *ATRX*. This is verified by the observed concurrence between kataegis and mutations in *TP53* and *ATRX* in African and European derived tumours. Notably, we previously found mutations in *TP53* and *ATRX* to be prominent features of the clinically adverse African-European specific molecular subtype (GMS-C)^10^, while shorter tumour telomere length has been observed in aggressive tumours from African men^14^. Given the dominance of SV-associated kataegis in European tumours, we extrapolate that the genesis of kataegis in European tumours may be driven by a SV-related mechanism, while in African tumour evolution, elevated APOBEC3 activity may be additionally implicated.

Our novel findings of kataegis features and mutational process between ancestries are dependent on our unique PCa whole genome data. The studied cohort remains the largest of its kind for the African continent, and benefits from the inclusion of clinically, technically and analytically matched non-African data, allowing for direct unbiased comparative analyses. The African inclusive data, further supported by published non-African data, enabled us to decern both universal (or shared) and ancestrally unique kataegis positive associated tumour features, particularly in advanced tumour disease. While overall kataegis was less common to African tumours, presence of independent kataegis may link to elevated off-targeted APOBEC3 activity, which may be responsible (at least in part) for the significant genomic and clinical heterogeneity observed for African men. Furthermore, our study emphasises the need for further African inclusion, Specifically to elucidate the potential for kataegis and APOBEC3 enzymes to be biomarkers of targeted cancer therapy. Collectively, by elucidating the manifestation of kataegis from tumorigenesis to later subclonal events in African and other ancestral patients, we highlight the significance of different underlying mutational progresses between ancestries which provide a valuable resource for targeted therapeutic interventions and emphasise the need for continued exploration of biological behaviours and environmental exposures of African patients.

## Methods

### Subjects and whole genome sequencing

Treatment naive samples of blood and tumour pairs were collected from 188 patients diagnosed with PCa recruited from South Africa (n = 109) and Australia (n = 79), with a bias towards more aggressive cases (78%, Table. 1). Two risk levels were defined by ISUP GG as HR including GG3–5 and LR including GG1 and GG2. All samples underwent deep WGS using the Illumina NovaSeq and Hiseq platforms, GRCh38 referenced variant calling and annotation, and evolutionary timing pipelines, as previously described^10^. Patient ancestry was determined using whole genome interrogation for subpopulation fraction analyses, as previously described^10^. In short, 109 patients categorised as African (all South African) have 86% African ancestral fraction; 57 were categorised as European (53 Australian, 4 South African), allowing up to 3% African ancestral and 26% Asian contributions^10^. The ancestry inference of 22 newly sequenced Asian patients (all Australians) was conducted using ADMIXTURE (v1.3.0)^37^ that compared 67,284 germline SNVs against subjects from the Human Genome Diversity Project (HGDP) and 1000 Genomes Project (1KGP), within gnomAD v3.1 database^38^. The best result was selected based on the optimal mean cross-validation (0.186) and nine out of ten replicates in concordance. Asian patients have over 89.6% Asian contributions.

### Public validation cohorts

Somatic SNVs were downloaded from published deep WGS tumour-normal data derived from 296 European and 207 Asian primary PCa donors, with available clinical data. In brief, while European data was derived from the Prostate Adenocarcinoma Canada (CA) project via the ICGC Data Portal^22, 23^, Asian data was obtained from Chinese Prostate Cancer Genome and Epigenome Atlas with Accession PRJCA001124^24^. While the European cohort is biased towards LR PCa, no age difference between LR and HR cases was observed for either the European (79%, n = 234 *vs* 21%, n = 62; median of age 64 *vs* 63.5, Wilcoxon’s rank sum test, *P*-value = 0.58) or Asian (35%, n = 73 *vs* 65%, n = 134, with same median of age 69). Kataegis analysis was performed as previously described.

### Kataegis identification from SNVs of evolutionary epoch

Kataegis identification applied the steps developed by PCAWG consortium^5^ in SNVs of the same evolutionary timing (detailed in Supplementary Methods). As kataegis is supposed to be resulted from a single mutational process, we called kataegis candidates from subsets of SNVs originated from the same evolutionary timing. The evolutionary timing of somatic SNVs was estimated with MutationTimeR^27^ and grouped into clonal (including early, late and unspecified) and subclonal epochs. For evolutionary kataegis candidates, we called kataegis from three subsets of SNVs for each genome, namely early clonal SNVs, late clonal SNVs, and subclonal SNVs. Unspecified clonal SNVs were included in both early and late clonal subsets. Unknown SNVs were included in all the three subsets. For each subset, inter-mutational distances of SNVs were adjusted with the piecewise constant fitting (PCF) model using the core algorithms of R package *kataegis*^39^, part of *copynumber* package^40^ with default parameters^17^. Following PCAWG steps^5^, threshold of calling kataegis was adjusted by the total number of SNVs per subset, which was a minimum of four SNVs with the PCF-adjusted inter-mutation less than 1 kbp. Overall, we identified 263 candidate kataegis in 68 patients.

Kataegis candidates were filtered with at least one of the two following kataegis characteristics^15, 17^: *i)* the consistency of SNV types in a kataegis event, and *ii*) residing on the identical chromosomal homolog/in *cis*. Most candidates (91%, 239/263) had consistent kataegic SNV types, while only 31% (82/263) showed available phasing information defined by phasing group IDs reported from GATK Somatic short variant discovery pipeline^41^. Twelve kataegis candidates were filtered out due to inconsistency of SNV types and lacking phasing information. As we included clonal unspecified SNVs in both early and late clonal SNV subsets, we observed two pairs of early clonal kataegis and late clonal kataegis sharing the same cluster of closely distributed unspecified SNVs. We manually removed those with less kataegic SNVs. After all, 249 kataegis events from 65 patients were chosen for downstream analyses.

### Kataegis identification for public validation cohorts

As evolutionary timing of SNVs was unknown for public cohorts, we applied the same method of kataegis calling on genome-wide SNVs. For the European cohort, we identified 960 candidates in 163 of 296 cases. While phasing information is not available, filtering is purely based on consecutiveness, resulting in 321 kataegis in 116 cases. For the Asian cohort, 378 candidates were identified in 127 of 207 cases. Again, phasing information is not available and as such filtering was based on consecutiveness, resulting in 297 kataegis in 103 cases.

### Statistical Analysis

We conducted statistical tests in R (v 4.2.2)^42^. Fisher’s exact tests were used to for comparison of two categorical variables using *stats* package^42^. Wilcoxon’s rank sum tests examined differences of continuous data between two ancestries or risk groups using *ggpubr* package (v0.6.0)^43^. *P*-values of multiple testings were adjusted using FDR with *Rstatix* package (v 0.7.2)^44^, specified in the results if adjusted. The significant threshold was 0.05 for *P*-values and FDRs. A single patient was regarded as an outlier due to extreme kataegis burden (42 kataegis events) while the median kataegis events was two for the other kataegis positive tumours (range, 1–12).

Generalised linear model was used to find the most contributing factors of kataegis. We used negative binomial regression model for kataegis burden as including many zero kataegis burden and with a variance greater than mean (4.03 *vs* 1.03). Three African patients with PSA or age unavailable and a European patient with hyper-kataegic tumour were excluded. The best model was selected with the optimal Akaike’s Information Criterion (AIC) in stepwise selection, from factors including age at diagnosis, risk level of the derived tumour, SV burden, ethnicity, CNV gain and loss, telomere lengths of blood and tumour. SV burden and region of CNV loss were log transformed for the adjustment of skewness. Although age distribution was different in our African cohort by risk-level group (median of age, HR = 69 *vs* LR = 64), age was not associated with the presence of kataegis, suggesting our findings on kataegis were unaffected by age bias. TMB was excluded as correlated with SV burden (correlation = 0.66). On the other hand, we used logistic regression model for kataegis presence with the same method of model selection and the same group of factors for model selection. SV burden and region of CNV loss were not log transformed as logistic regression model had no assumption for distribution of contributing factors. The best selected model for African and European patients included ethnicity, although not significant (*P*-value = 0.07). As ethnicity may be associated with kataegis presence, at least improve the fitting model of kataegis presence, performed the model selection on the African and European patients separately with the same method.

### Survival outcomes

We performed survival analyses of European and Asian patients with HR tumours and with extensive follow-up data. Kaplan-Meier estimates were conducted using *survival* package (v 3.5–5)^45^ in R. The hyper-kataegic tumour was regarded as outlier, excluded from the analysis. Cancer progression defined by BCR and/or metastasis was plotted with the follow-up time in months. Additionally, we compared the survival distribution with progression defined by having bone metastasis, excluding BCR patients without bone metastasis. We analysed log-rank tests of survival distributions using *survminer* package (v 0.4.9)^46^ between kataegis positive and negative, and between patients with multiple kataegis events (kataegis count > 1) and the others (kataegis count = 0, 1). Survival analyses were also performed for the 284 public European patients with available follow-up data. The susceptibility of patients with multiple kataegis events to BCR and/or metastasis, and metastasis were evaluated within LR and HR tumours groups.

### SBS and SV signatures

To study biological processes underlying kataegis, and any differences in biological processes between kataegis positives and negatives, kataegic SNVs and genome-wide SNVs were decomposed and assigned with the conventional SBS signatures using SigProfilerExtractor (v.1.1.22)^47^. SBS signatures of kataegic SNVs was made from combining the studied cohort (excluding for the single outlier), public European, and public Asian cohorts together. Three public European patients were further excluded due to high TMB and absolute z-score greater than three. Kataegic SNVs from the public European data was changed to GRCh38 reference using liftOver^48^. The initial step extracted de novo signatures with nonnegative matrix factorisation (NMF) from the matrix of frequencies of 96 SBS classes that are defined by six substitutions (C > A, C > G, C > T, T > A, T > C, and T > G) and their 5’ and 3’ nucleotide context. After the extraction, the automatically selected optimal set of signatures were matched/assigned to the set of conventional COSMIC signatures (Catalogue of Somatic Mutations in Cancer v3.4, Oct. 2023). We used default settings with modification on some parameters including 15 maximum signatures, 500 NMF replicates, 1 million maximal NMF iterations, and GRCh38 reference. From kataegic SBS signatures results, 31 tumours were filtered for cosine similarity lower than 0.5. From resulted SBS signatures, we used Wilcoxon’s rank sum tests to compared rates of APOBEC-related signatures against other aetiologies, and rates of SBS2 against the total APOBEC-related signatures between HR and LR tumours for each ancestry. Pearson correlations of APOBEC signature rates and kataegis load were estimated using R stats package (v 4.2.2).

For SV signatures and their biological processes, NMF extraction methods were based on the frequency matrix of 32 SV types^29^. Besides translocation types, the matrix consisted of deletions, inversions, and tandem duplication types with five size ranges. SVs were also categorised into clustered and non-clustered based on distance of two adjacent SVs. The same SigProfilerExtractor parameters and version of conventional COSMIC SV signatures as described above were applied. The SV signatures were analysed for both genome-wide SVs and those proximal to kataegis events (1kb distance from SV breakpoints). For kataegis positive *versus* negative genomes, Wilcoxon’s rank sum tests compared the rate of SV2 against other signatures. SV signatures, SV4 and SV10 were of low proportion, so Fisher’s exact test was performed to compare their kataegis prevalence’s.

### Enrichment of kataegis around proximal SVs

To verify the two enrichments of kataegis around 1 kbp and 1Mbp to proximal SVs in HR tumours, we compared distances to proximal SVs of kataegis against simulated random regions as background. The comparison using Fisher’s exact test included HR tumours only and excluded a hyper-kataegic tumour as an outlier. To make a comparable simulation, for each kataegis of 182 events identified from HR tumours, we randomly selected a somatic small variant from the same tumour, excluding HLA and ALT regions, and assigned the selection with the same range of the spanning of the kataegis event. For the investigation of enrichment of kataegis around 1 kbp of SVs, we compared the number of kataegis within 10 kbp region of SV breakends against the number located outside the 10 kbp region of SVs breakends against those numbers observed in the simulations. Likewise, the investigation of enrichment of kagtaegis around 1 Mbp of SV breakends was to compare the number of kataegis with distance range between 0.1Mbp and 10Mbp of SV breakends against those with distance more than 10Mbp, against those in simulations.

## Figures and Tables

**Table 1:** Demographic and clinical information of the studied cohort

Ancestry	Patients (n)	Country		Risk level		Median age (range)
		South Africa	Australia	Low-risk (GG1/2)	High-risk (GG3–5)	
African	109	100% (109)	0	26% (28)	74% (81)	68 (45–99)*
European	57	7% (4)	93% (53)	12% (7)	88% (50)	63 (46–72)
Asian	22	0	100% (22)	27% (6)	73% (16)	69.85 (51.6–77.8)
**Total**	**188**	**60% (113)**	**40% (75)**	**22%** (41)	**78% (147)**	**66 (45–99)**

* An African patient has missing age information.

## Data Availability

Published sequence data were obtained through the European Genome‐Phenome Archive (EGA; https://ega‐archive.org) under overarching accession EGAS00001006425 for the SAPCS (EGAD00001009067) and Garvan/St Vincent’s Dataset (EGAD00001009066) and for the later including new unpublished data for 22 Asian ancestral genomes.

## List Of Abbreviations

1KGP: 1000 Genomes Project
AFR: African
AIC: Akaike’s Information Criterion
APOBEC3: apolipoprotein B mRNA editing catalytic polypeptide-like 3
ASI: Asian
BCR: biochemical relapse
BIR: break induced replication
CA: Canada
CGC: Cancer Gene Census
CNV: copy number variants
DSB: double-strand breaks
EUR: European
GG: Grade group
GMS: global mutational subtypes
HGDP: Human Genome Diversity Project
HR: high-risk
ICGC: International Cancer Genome Consortium
ISUP: International Society of Urological Pathology
LR: low risk
NMF: nonnegative matrix factorisation
PCAWG: Pan-Cancer Analysis of Whole Genomes
PCF: piecewise constant fitting
PCa: Prostate cancer
PSA: prostate-specific antigen
SAPCS: The Southern African Prostate Cancer Study
SBS: single-base substitution
ssDNA: single strand DNAs
SNV: single nucleotide variants
SV: structural variants
TCGA: The Cancer Genome Atlas
TMB: tumour mutational burden
WGS: blood whole genome sequencing
